# Detection and genotyping of CMV and HPV in tumors and fallopian tubes from epithelial ovarian cancer patients

**DOI:** 10.1038/s41598-019-56448-1

**Published:** 2019-12-27

**Authors:** Edyta Paradowska, Agnieszka Jabłońska, Mirosława Studzińska, Miłosz Wilczyński, Jacek R. Wilczyński

**Affiliations:** 1 Laboratory of Virology, Institute of Medical Biology of the Polish Academy of Sciences, Lodz, Poland; 2 Department of Surgical, Endoscopic and Oncological Gynecology, Polish Mother’s Health Center Research Institute, Lodz, Poland; 3 Department of Surgical and Oncological Gynecology, Medical University of Lodz, Lodz, Poland

**Keywords:** Tumour virus infections, Virology

## Abstract

Viral and bacterial infections are detected in epithelial ovarian cancer (EOC) tissues. Since the fallopian tubes are often affected by pelvic inflammatory disease (PID) and the majority of serous EOCs appear to originate from dysplastic lesions in the distal tube, it is relevant to consider the potential role that infectious agents may play in ovarian carcinogenesis. We sought to analyze the prevalence of human papillomavirus (HPV) and cytomegalovirus (CMV) in EOC tissue and fallopian tube specimens obtained at tumor resection. Ovarian cancer and fallopian tube tissue samples obtained from patients with EOC were analyzed by both qualitative and quantitative PCR to detect and quantify viral DNA. The presence of CMV and HPV DNA was detected in 70% and 74% cancerous ovarian tissues, respectively, and was significantly higher in EOC than in benign tumor cases (*P* ≤ 0.01). CMV or HPV infection was observed also in the fallopian tube samples. Infection with HPV16 was determined in 70% of EOC cases. Almost two thirds of EOC patients demonstrated coinfection with CMV and HPV in the pathological samples. The results revealed that the presence of CMV and HPV in EOC samples is common. CMV and HPV infections can be potential risks for EOC development.

## Introduction

Ovarian cancer is the leading cause of cancer-related deaths in women, accounting for almost 300,000 new cancer cases, and 185,000 deaths in 2018 worldwide^1^. It has nonspecific symptoms, causing more than 60% of cases to be diagnosed at the advanced stage^2^. Ovarian cancer is a highly fatal disease, with a global 5-year age-standardized survival rate of 30–40% in women at advanced stages of diagnosis^3,4^. Approximately 90% of all ovarian cancers have epithelial origins and these cancers are more invasive^5^. Ovarian cancer is a heterogeneous disease that can be divided into three most common types: epithelial ovarian cancer (EOC), germ cell tumors, and sex cord-stromal tumors. The most common EOC histologic subtypes are serous, mucinous, endometrioid, clear-cell, or any combination of these subtypes^6^. The various histotypes are associated with distinct molecular alterations with different etiology, epidemiology, treatment, and prognosis. High-grade serous ovarian carcinoma (HGSOC) is the most aggressive and common subtype of EOC, representing approximately 75% of EOC cases^7^. EOCs originate from the ovarian surface epithelium and from serous tubal intraepithelial carcinomas (STICs) from the fallopian tube epithelium^8–11^. The fimbriae of the fallopian tube, which are in close proximity to the ovarian surface, have been suggested as the primary site of HGSOC origin^12^. The presence of lesions in the fallopian tube that have cytological features similar to those observed in HGSOCs is designated as STIC^13^. STICs harbor clonal mutations in the *TP53* gene encoding the tumor suppressor p53, indicating that these molecular alterations are as an early event in the oncogenesis of HGSOCs^14,15^. A large and growing body of literature supports a theory that STIC is a precursor of HGSOC involving uterine adnexa and peritoneum^16^.

More than one-fifth of ovarian carcinoma appears to be associated with inherited risk, including mutations, e.g., mutations in the *BRCA1/2 and TP53* genes^17,18^. Among other factors, such as hereditary, environment, and lifestyle, chronic inflammation seems to be an important risk factor for EOC development. Epithelial cells are exposed to increased levels of inflammatory mediators including pro-inflammatory cytokines, chemokines and hormones that induce DNA damage *via* oxidative stress and contribute to increased cell division, genetic and epigenetic changes. The main causes of inflammation in the ovaries and fallopian tubes are ovulation, infection, and endometriosis. The predominant hypothesis on ovarian carcinogenesis suggests that incessant ovulation, ovarian rupture and inflammation cause a pro-oxidative microenvironment and mutagenic DNA damage^19,20^. Pelvic inflammatory disease (PID), an infection of the female reproductive organs, also results in DNA damage and a pro-inflammatory response. The role of environmental factors such as bacterial (e.g., *Chlamydia trachomatis*, *Mycoplasma genitalium)* and viral infections is still under investigation^21–25^. Recently, it has been shown that *C*. *trachomatis* DNA is highly expressed in tubal serous cancer compared with high-grade serous ovarian cancer^26^. Some reports confirmed the presence of human papilloma virus (HPV) in malignant ovarian cancer^25,27–34^, while others did not^35–38^. High-risk HPV (HR-HPV) types 16 and 18 were the predominant genotypes associated with advanced stages^28,30,31,34,39,40^. However, the low-risk HPV (LR-HPV) type 6 was also detected^25^. The HR-HPV E6 and E7 oncoproteins can inactivate the tumor suppressors’ p53 and Rb, respectively^41^. Evidence of cytomegalovirus (CMV) and/or Epstein-Barr virus (EBV) infection in ovarian cancer tissues was also detected^25,42–44^. These widespread herpesviruses can establish life-long infections and contain oncoproteins promoting malignant transformation and metastasis. CMV can spread to the upper genital tract and persistent infection is usually asymptomatic. The oncomodulatory properties of CMV may play an important role in ovarian carcinogenesis and disease progression.

To elucidate the potential role of viruses in ovarian carcinogenesis, the prevalence of HPV types and herpesviruses in EOC and fallopian tube tissue samples was determined. Variation in the *UL55* and the *US28* genes was determined in all patients with active CMV infection.

## Results

### Study population

The tissue material from patients with EOC consisted of 20 HGSOCs, four adenocarcinomas (mucinous or clear-cell), and other cell neoplasms. According to the FIGO stage distribution, two patients had stage I, one patient had stage II, 20 had stage III, and 4 had stage IV. In addition, four metastatic tumors and eight benign tumors as control group were included in the study. The average age at which the EOC patients were examined was 63.3 years (median age 66.0 years; range 34–85 years), while in the control group, it was 55.0 years (median age 53.5 years; range 37–88 years). The demographic and clinical characteristics of the patients are summarized in Table 1.

**Table 1:** Patient characteristics.

Patient characteristics	
**Epithelial ovarian cancer** (n = 27)	
**Median age**, years (range)	66.0 (34–85)
**Tumor histology**	n (%)
HGSOC^a^	20
Other types	7
BOT^b^	2
Mucinous adenocarcinoma	2
Clarocellular adenocarcinoma	2
Undifferentiated carcinoma	1
FIGO stage^c^	n (%)
I	2 (7.4%)
II	1 (3.7%)
III	20 (74.1%)
IV	4 (14.8%)
**Metastatic ovarian cancer** (n = 4)	
Gastric cancer	1
Colon cancer	1
Breast cancer	1
Undetermined primary site	1
**Benign tumors (n** = **8)**	
**Median age**, years (range)	53.5 (37–88)
**Tumor histology**	n
Uterine fibroids	5
Serous ovarian cyst	1
Mucinous ovarian cyst	1
Dermoid ovarian cyst	1

^a^HGSOC – high-grade serous ovarian cancer; ^b^BOT – serous border-line ovarian tumor;

^c^The International Federation of Gynecology and Obstetrics (FIGO) clinical stage.

### Detection of herpesvirus infection

**Figure 1:**
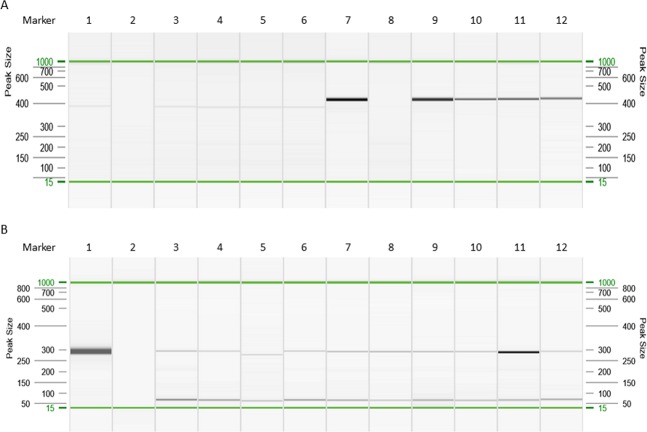
Visualization of PCR products for CMV *US28* and *UL55* genes (**A**), and HPV16 *E6* gene (**B**). Gel image: CMV *US28* gene: 1–6, CMV *UL55* gene: 7–12. 1,7. Positive control (CMV AD-169); 2, 8. Negative control; 3, 9. Blood; 4, 10. Ovarian cancer; 5,11. Fallopian tube; 6, 12. Primary ovarian cells. B. Gel image: HPV16 *E6* gene. 1. Positive control (Ca Ski cells); 2. Negative control; 3, 4. Blood; 5–8, Ovarian cancer; 9, 10. Fallopian tube; 11, 12. Primary ovarian cells. Alignment markers (15 bp, 1 kbp).

DNA isolated from blood, primary cancer cells, tumors, and fallopian tube samples was examined by nested PCR and quantitative real-time PCR (qRT- PCR) assays for the detection of CMV, EBV, and HSV-1. CMV infection was confirmed on the basis of CMV DNA detection (Fig. 1) and the results of serological assays. The CMV viremia was confirmed in 16/27 (59.3%) patients with EOC, all patients with metastatic tumors, and in 3/8 (37.5%) individuals with benign tumors. CMV DNA was detected in 19/27 (70.4%) of ovarian cancer samples and in all tissue samples obtained from patients with metastatic cancer. In the control patients with benign cancers, all ovarian samples were negative. The frequency of CMV DNA detection was significantly higher in EOC than in benign tumor cases (odds ratio, OR 39.00; 95% confidence interval, CI: 2.01–756.00; *P* = 0.0005). The prevalence of the viral infections is summarized in Table 2. In addition, CMV DNA was detected in three samples of the fallopian tube derived from patients with EOC and in one sample obtained from a patient with metastatic adenocarcinoma. Among the 20 patients with HGSOC, CMV DNA was found in 14 (70.0%) of ovarian cancer specimens. Sequence analysis of the PCR products revealed that 18/19 (94.7%) of CMV-positive patients with EOC were infected with the CMV gB2 (*UL55*) genotype and one with the gB5 genotype. In all cases, the *US28* A2 genotype of CMV was found. These CMV genotypes occurred also in patients with metastatic cancer.

**Table 2:** The prevalence of CMV and HPV types in tissue materials from patients with epithelial ovarian cancer, metastatic tumors and benign tumors.

Virus	Prevalence; n (%)				
	EOC	Benign tumors	*P*^*a*^ value	Metastasis	*P*^*b*^ value
CMV	19/27 (70.4)	0/8 (0)	**0**.**0005**^*c*^	4/4 (100)	0.550
HPV6	2/27 (7.4)	0/8 (0)	1.000	0/4 (0)	1.000
HPV16	19/27 (70.4)	2/8 (25.0)	**0**.**039**^*d*^	4/4 (100)	0.550
HPV18	0/27 (0)	0/8 (0)	1.000	0/4 (0)	1.000
HPV45	1/27 (3.7)	0/8 (0)	1.000	0/4 (0)	1.000
CMV/HPV6	2/27 (7.4)	0/8 (0)	1.000	0/4 (0)	1.000
CMV/HPV16	16/27 (59.3)	0/8 (0)	**0**.**004**^*e*^	4/4 (100)	0.269
CMV/HPV45	1/27 (3.7)	0/8 (0)	1.000	0/4 (0)	1.000
EBV	0/24 (0)	0/8 (0)	1.000	0/4 (0)	1.000
HSV-1	0/24 (0)	0/8 (0)	1.000	0/4 (0)	1.000

n, number of cases with the virus genotype; *P*, Fisher’s exact test was used to compare virus distribution between the patient groups with EOC, benign tumors and metastasis; ^*a*^the significance *P*-values between prevalence in EOC and benign tumor samples; ^*b*^the significance *P*-values between prevalence in EOC and metastatic samples; ^*c*^odds ratio (OR) 39.00, 95% confidence interval (CI) 2.01–756.00; ^*d*^OR 7.12, 95% CI 1.18–43.16; ^*e*^OR 24.39, 95% CI 1.28–466.30.

**Figure 2:**
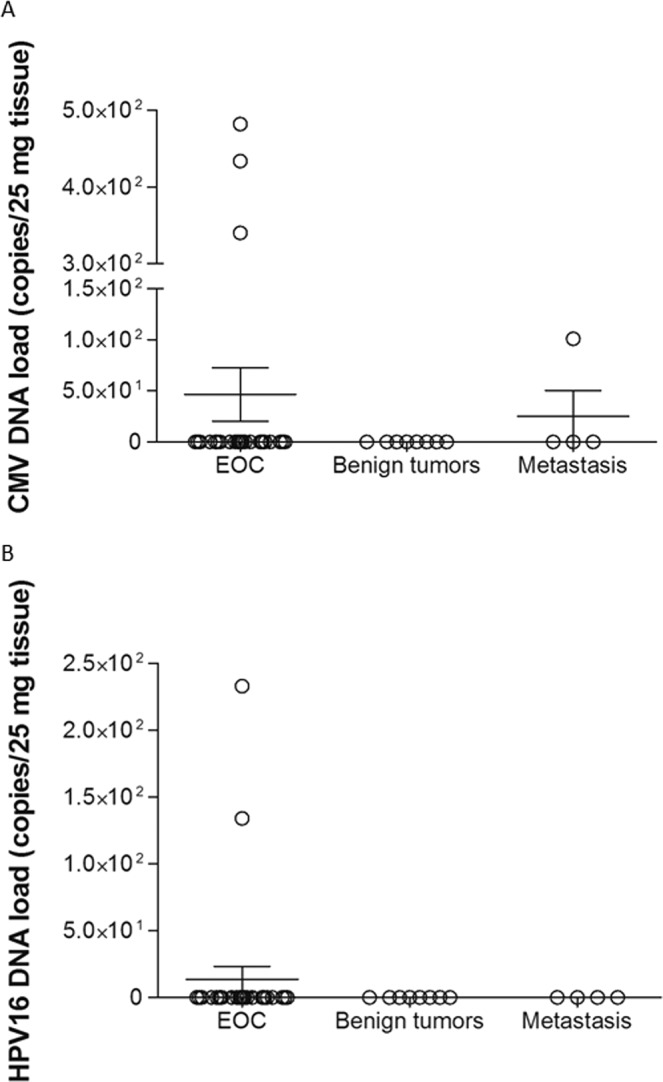
CMV (**A**) and HPV16 (**B**) copy numbers in ovarian tissue samples from EOC cases and patients with benign tumors or metastasis. The CMV and HPV16 copy numbers were quantified using a qRT-PCR method with the genesig Standard Kits (Primerdesign Ltd.). The sensitivity of the assays has been determined to be approximately 100 copies of target template. Bars in the scatter dot plot represent the mean viral loads and whiskers represent the SEM values.

A real-time PCR assay was used to quantify CMV DNA levels in blood and tissue samples. CMV DNA was detected in two of the 27 EOC samples (7.4%) using the genesig Standard Kit. The mean CMV DNA concentration in infected tissues was 3.59 × 10^2^ copies/25 mg; range 3.13 × 10^2^–4.35 × 10^2^ copies (Fig. 2A). Serum samples collected from patients with EOC were CMV- IgM and IgG positive in 61.5% and 92.3% cases, respectively. None of the tumor and blood samples were positive for EBV or HSV-1 DNA based on qRT-PCR and PCR (EBV) analysis.

### Detection of HPV types

We examined the prevalence of HPV genotypes in tumor tissue samples by polymerase chain reaction. HPV genotypes determined by PCR were sequenced, and no discrepancies were found. HPV DNA was detected in approximately 74.1% (20/27) of EOC tissue samples (Table 2). The most common HPV types found in EOC samples were HPV16 (19/27, 70.4%), followed by HPV6 and HPV45. Two patients were positive for HPV6 (cancer or fallopian tube samples), whereas ovarian sample from one patient was infected with HPV45. In addition, HPV16 DNA in all cases of metastatic tumors and in 25.0% (2/8) benign tumors was detected. The HPV16 DNA was detected more frequently in tissues from patients with EOC compared to benign tumors (OR 7.12; 95% CI: 1.18–43.16; *P* = 0.039). The significance of these results must be handled with caution due to small sample size in both patient groups. In addition, HPV16 DNA in the blood samples from 7/27 (25.9%) patients with EOC and 2/4 (50.0%) metastatic tumors has been detected. HPV infection was observed in the fallopian tube samples of five patients with EOC (HPV16 in four cases and HPV6 in one case) and three patients with metastasis (HPV16). Analysis of specimens obtained from patients with HGSOC showed that HPV16 DNA was detected in 14/20 (70.0%) EOC samples.

Using qRT-PCR assays, high-risk HPV16 was detected in two samples of EOC (2/27, 7.4%). The HPV16 DNA concentration was low (1.34 × 10^2^ copies and 2.33 × 10^2^ copies/25 mg; Fig. 2B). None of the samples analyzed for HPV subtypes 18, 31, 33, 35, 39, 45, 51, 52, 56, 58, 59, 66 and 68 tested were positive.

Coinfection with both CMV and HPV was observed in samples obtained from seventeen patients with EOC (63.0%), including 16 cases with CMV and HPV16 infection (one case with CMV, HPV6 and HPV16), and one case with CMV, HPV6 and HPV45 infection (Table 2). Moreover, the frequency of CMV and HPV16 coinfection was significantly higher in EOC than in benign tumor cases (*P* = 0.004; Fisher’s exact test). Univariate analysis revealed that the odds of being coinfected were 24 times greater for cases with EOC than those with benign tumor (OR 24.39; 95% CI 1.28–466.30).

## Discussion

In this study, the presence of CMV and/or HPV was detected both in cancer and fallopian tube DNA isolated from patients with EOC. Among the examined patients, especially those with HGSOC, CMV and HPV DNAemia was common and was detected in more than two thirds of patients. Furthermore, more than half of the patients with EOC were coinfected with both CMV and HR-HPV. To our knowledge, this is the first demonstration of HPV/CMV coinfection of the upper genital track and CMV infection of the fallopian tubes in patients with EOC. Low amounts of viral DNA copies indicate that CMV and HPV may be present at low activity in EOC tissues. These results suggest that CMV exists in ovarian and fallopian tube cells in a latent phase and could be reactivated under the influence of the inflammatory tumor microenvironment. Extremely low amounts of viral DNA copies and increased levels of specific IgM antibodies found in the majority of patients confirmed our hypothesis. CMV is suspected to act as an oncomodulator that may infect cancer cells and modulate their malignant properties in a way not involving direct transformation^45,46^. Moreover, CMV infection elicits biological responses that are similar to those in chronic inflammation, leukocyte dysfunction, and angiogenesis. CMV coinfection can promote HPV-induced transformation and cause a more rapid development of cancer. Hence, we propose that coinfection with HR-HPV and CMV may be a risk factor for EOC development.

HPV is a carcinogenic virus and a major cause of cervical, anal, penile, and oropharyngeal cancer. A meta-analysis revealed an association between HPV infection and ovarian cancer, as the virus was detected in 17.5% of cases^47^. A geographical discrepancy in HPV prevalence in EOC tissue was found. HPV was detected in 15.5–17.0% of OC patients in North America, 4.0–18.5% in Europe, and 31.4–45.6% in Asia^25,33,39,47–49^. HPV does not play an important role in Western European and North American populations^48^. Although more than 200 different HPV types have been characterized, HPV types 6, 11, 16 and 18 are the most clinically relevant. HPV types 6 and 11 account for approximately 100% of genital wart cases, while 20–50% of lesions also contain coinfections with HR-HPV types^50^. Generally, HPV16, 18, 6, 33, and 45 genotypes were found in ovarian cancer. This study confirmed the common presence of HPV16 in 70% of EOC samples, including ovarian serous carcinomas. HPV16 was the most common genotype followed by HPV18 in other studies, although HPV18 was also identified^25,30,33,40,51,52^. HPV16 and/or HPV18 were detected not only in OC tissue, but also in fallopian tubes^53^.

HPV is assembled in the superficial layers and replicates without releasing virions into the bloodstream, and thus there is minimal immune recognition by the host. HPV reaches the ovarian epithelium and can be integrated into the genome of EOC cells^24,48^. This results in increased expression of viral E6 and E7 oncoproteins that inactivate two cellular tumor suppressor proteins, p53 and Rb, and leads to increased cell proliferation and malignant transformation. LR-HPV E6 and E7 proteins have lower binding affinities for tumor suppressor proteins compared with those of HR-HPV types^54,55^. Both oncoproteins target the cell cycle regulators, causing suppression of cell apoptosis that leads to extension of cell life span and increase in HPV replication^56^. Moreover, continuous expression of E6 and E7 in HPV-positive cancer cells is linked to significant alterations in the amounts of intracellular and exosomal miRNAs that are linked to the regulation of cell proliferation, senescence and apoptosis^57^.

The present investigation showed positive amplification in approximately 70% of EOC samples of two CMV genes, *UL55* encoding envelope gB and *US28* that encodes chemokine receptor US28. Sequence analysis revealed that the CMV gB2 and pUS28 A2 genotype was prevalent in EOC cases, which is in agreement with the genotype distribution in infants and adults from Central Poland^58,59^. Our findings are compatible with the results of Radestad *et al*., who found the expression of CMV immediate-early (IE) protein in 75% cases, while late CMV tegument protein (pp65) in 67% of ovarian cancer cases in the Gotland (Swedish) population^43^. The results by Shanmughapriya *et al*. showed the presence of low amounts of CMV DNA in 50% of tumor tissue samples obtained from women with EOC, while low protein expression was found in the majority of tissue sections, including those from patients with serous EOC^25^. We found that active CMV infection may occur both in EOC tissues and fallopian tubes. Earlier studies have shown that CMV infection of the fallopian tubes may be associated with ectopic pregnancy^60^. It should be noted, that CMV infection was low-grade and was only detected by employing sensitive techniques such as nested PCR.

CMV is a widespread opportunistic pathogen that is carried by 40–100% of the world’s population, depending on age, socioeconomic status, and geographical location^61^. CMV has developed mechanisms that allow it to survive in latent form in an immunocompetent host, while it reactivates during immunosuppression. CMV can spread to the upper genital tract, and infection is usually persistent, latent and asymptomatic. Several studies have identified high frequency of active CMV infection in tumor tissues, including colorectal cancer^62,63^, malignant glioma^64^, prostatic neoplasia and carcinoma^65^, cervix cancer^66^, and EBV-negative Hodgkin’s lymphoma^67^. Recently, CMV DNA and proteins were detected in ovarian cancer, including HGSOC, and in benign cystadenomas^25,43,44^. CMV is not considered to be oncogenic but is rather oncomodulatory in nature, although the mechanisms of its contribution to cancer remain poorly understood^68–70^. CMV is regarded as an oncomodulator because it promotes cell proliferation, and cell-cell progression, vascular disease development, and immune evasion that contribute to the development of autoimmune diseases and inhibit apoptosis^69,71^. CMV infection may thus promote malignant transformation by controlling of the cell cycle and dysregulating of physiological processes. The CMV *US*28 gene was shown to be a key mediator of virus-induced vascular disease and has been implicated in models of CMV-associated glioblastoma^70,72^. The US28 protein activates NF-ĸB, which directly transactivates the CMV major IE promoter^73^ and induces caspase-dependent apoptosis in different cell lines^74^. This signaling pathway probably plays a role in CMV-mediated inflammatory diseases and CMV-mediated oncogenesis^69^. CMV encodes four proteins, IE1, IE2, pp71, and pUL97 that can bind or phosphorylate Rb family proteins and inhibit the cell cycle arrest functions of p53. Moreover, CMV induces a mesenchymal-to-epithelial transition (MET)^75^. In contrast, EBV DNA was occasionally detected in EOC tissue (5% of cases)^42,76^, and no association between EOC and EBV antibody levels was found^23^. Taken together, these results indicate that the role of the EBV in ovarian malignancy is probably less important than that of CMV.

The main limitation of this study was the small sample size and the limited availability of some clinical materials; therefore, HPV and CMV positivity might not reflect a true statistical distribution. Further studies using larger patient groups are needed to confirm our findings. Despite a limited number of specimens in this study, our findings may indicate an important role for HPV, CMV and coinfection with both in the development of ovarian cancer.

In conclusion, HPV is probably responsible for some cases of ovarian cancer, while CMV may act as oncomodulatory virus and may promote disease progression. A detected coinfection of CMV and HPV in ovarian cancer and/or fallopian tube indicates a potential oncogenic interplay between the two viruses. A randomized control trial is needed to clarify whether anti-viral therapy is beneficial to EOC patients with CMV and/or HPV detected in the tumor. The results may provide new insight into the pathogenesis of ovarian cancer and new strategies for the use of antiviral therapy in oncology patients.

## Methods

### Study design and participants

Between November 2017 and June 2019, 27 women aged 34–85 years with presumed epithelial ovarian cancer were enrolled in the study. The inclusion criterion was referral for surgery to a specialized center on suspicion of EOC. Women were considered ineligible to participate if they met any of the following criteria: synchronous cancer other than EOC and ovarian cancer of nonepithelial origin. Patients underwent cytoreductive surgery at the Department of Surgical and Oncological Gynecology, Medical University of Lodz, or at the Tomaszow Health Center, Poland performed by the same experienced oncological gynecologist. After a midline longitudinal incision of the abdominal wall, meticulous inspection of the peritoneal cavity was performed, and the tumor was excised for intraoperative pathological examination. Samples of both the tumor tissue and distal part of the fallopian tube (including tubal fimbriae) unilateral to the tumor site were obtained and immediately secured for laboratory tests after confirmation of EOC by a pathologist. Then, cytoreductive surgery was performed with the intention to obtain a complete cytoreduction protocol. The control group consisted of 8 patients aged 37–88 years who underwent surgery for uterine fibroids or benign ovarian tumors. Finally, the tissue material consisted of 20 HGSOCs, 7 ovarian cancers of other histologic types, and 4 metastatic ovarian cancers. Because metastatic ovarian cancer is histologically different from primary ovarian cancer, the 4 patients with metastatic tumors were excluded from the study group and incorporated into a distinct group for comparison. The basic tumor characteristics are presented in Table 1. Tumor and blood samples from patients with benign ovarian cystadenoma were included as a control group. All diagnoses were confirmed by gynecological pathologist at the Department of Pathology, either of the Medical University or the Polish Mother’s Health Center Research Institute, Lodz, Poland. All subjects were of European descent, and there were no ethnic differences between the EOC cases and the control group.

The study protocols were approved by the Bioethics Committee of the Medical University of Lodz (RNN/346/17/KE from 21 November 2017). All procedures performed in the studies were in accordance with the Helsinki declaration. All patients provided written informed consent for participation in this study.

### Tissue collection and cultures of primary ovarian cancer cells

Tumor and blood samples were obtained from 27 patients with EOC, 4 patients with metastatic cancer, and 8 patients with benign tumors. In addition, fallopian tube tissue samples were obtained from 10 females diagnosed with EOC and 3 women with metastatic cancers. Tumor and fallopian tube tissue samples were collected at the time of surgery in ice-cold Dulbecco’s Phosphate Buffered Saline (DPBS; Sigma-Aldrich Co. Ltd., Ayrshire, UK) and processed within 1 hour. The solid ovarian cancer specimens were washed with ice-cold DPBS to remove contaminating blood and then minced into small pieces (approximately 1–2 mm^3^). Primary ovarian cancer cells were isolated from solid specimens using the modified method described by Pribyl *et al*.^77^. The tissue pieces were digested with 150 U/mL collagenase (Sigma-Aldrich, St. Louis, MO, USA) for 30 min at 37 °C and then transferred onto a nylon cell strainer (100 µm; VWR International, Radnor, PA) to separate EOC cells. The cultures were maintained at 37 °C in 5% CO_2_ for several days. EOC cells form a confluent monolayer after two weeks in culture. The cells were collected by centrifugation and stored at −80 °C until use.

### Cell lines and virus strains

MRC-5 (ATCC CCL-171, American Type Culture Collection, Manassas, VA, USA), HeLa (ATCC CCL-2), and Ca Ski (ATCC CRL-1550) cells were cultured in Eagle’s Minimum Essential Medium (EMEM; Sigma-Aldrich) or RPMI-160 (Sigma-Aldrich), respectively, supplemented with 10% inactivated fetal bovine serum (FBS), 2 mM L-glutamine, and 100 μg streptomycin-100 U penicillin at 37 °C in a 5% CO_2_ atmosphere until confluent. HeLa cells contain HPV-18 sequences, whereas Ca Ski cells contain an integrated HPV16 genome as well as sequences related to HPV18. The CMV strain AD-169 (ATCC VR-538) was propagated in MRC-5 cell line in EMEM supplemented with 2% inactivated FBS, L-glutamine, and antibiotics.

### CMV serology

Serum samples from the patients were assessed for anti-CMV IgG and IgM antibodies with the use of NovaLisa CMV IgM and IgG assays (NovaTec Immundiagnostica GmbH, Dietzenbach, Germany) based on the enzyme-linked immunosorbent assay (ELISA).

### DNA extraction

Genomic DNA isolation from tissue specimens and blood was performed using a QIAamp DNA Mini Kit and Blood Mini Kit (Qiagen GmbH, Hilden, Germany) according to the manufacturer’s instructions. The concentration and purity of DNA were assessed using a NanoDrop 2000c UV-Vis Spectrophotometer (Thermo Scientific, Waltham, MA, USA). DNA was extracted from 200 µl of blood, 5 × 10^6^ cancer cells, and 25 mg tissue, eluted in 100 µl of elution buffer, and then stored at −20 °C.

### Detection and assessment of CMV DNA

The CMV DNA was quantified using a qRT-PCR method with the genesig Standard Kit (Primerdesign Ltd, York House, UK) according to the manufacturer’s instructions. The DNA corresponding to the CMV *UL55* gene was quantified using the 7900HT Fast Real-Time PCR system (Applied Biosystems, Foster City, CA, USA). To validate any positive findings, a negative control without template DNA or RNA was included in each run.

The hypervariable fragments of the CMV genes, *UL55* and *US28*, were amplified with DreamTaq DNA polymerase (Thermo Fisher Scientific, Carlsbad, CA, USA) using a nested and heminested PCR method, as described previously^58^. DNA isolates from MRC-5 cells infected with CMV laboratory strain AD-169 was used as a positive control, and nuclease-free water was used as a negative control. Positive and negative controls were included with each run. All amplifications were carried out in a Veriti 96-Well Thermal Cycler (Applied Biosystems). The amplicons were separated and analyzed using the QIAxcel system (Qiagen). The retention time of the PCR fragments relative to the QX Alignment Marker 15 bp/1 kb fragments was calculated using the BioCalculator software (Qiagen). The PCR product sizes were determined by comparing the retention time with the QX DNA Size Marker 50–800 bp. The PCR products were sequenced using the MiSeq system (Illumina, San Diego, CA, USA). The obtained sequences were analyzed and verified with the Chromas-Win95/98/NT/2000/XP and Basic Local Alignment Search Tool (BLAST). The results were compared with reference sequences in the GenBank database.

### Detection of other herpesviruses

The human DNA herpesviruses, including Epstein-Barr virus (EBV), and herpes simplex type 1 (HSV-1) were quantified using a qRT-PCR method with the genesig Standard Kits (Primerdesign Ltd.) as described above. The PCR test has been used for detection of EBV DNA in specimens as described previously^78^.

### Detection and assessment of HPV DNA

The HPV16 and HPV18 copy numbers were quantified using a qRT-PCR method with the genesig Standard Kits (Primerdesign Ltd.) as described above. The test amplifies the region that codes HR-HPV E6 oncoprotein. High risk Human Papillomavirus Multiplex screening genesig kit (Primerdesign Ltd.) was used to simultaneously detection of DNA of 14 HPV types (16, 18, 31, 33, 35, 39, 45, 51, 52, 56, 58, 59, 66, and 68). The DNA was quantified using the LightCycler 96 System (Roche Diagnostics GmbH, Mannheim, Germany). AmpliSens HPV16/18-FRT PCR kit was used for qualitative and quantitative detection and differentiation of HPV types 16 and 18 DNA using real-time hybridization-fluorescence detection of amplified products (InterLabService Ltd., Moscow, Russian Federation). The HPV DNA was quantified using the 7900HT Fast Real-Time PCR system (Applied Biosystems).

The uniplex PCR tests with HPV-type specific primers for *E6* gene were used for detection of the HPV6, 16, 18, and 45 types^79^. The amplified products were the following: 291 bp (HPV6), 274 bp (HPV16), 224 bp (HPV18), and 282 bp (HPV45). PCR was performed in a Veriti 96-Well Thermal Cycler (Applied Biosystems). The conditions for amplification with all primers sets were 95 °C for 5 min, 35 cycles at 95 °C for 30 sec, 60 °C for 30 sec, and 72 °C for 60 sec, and a final 5 min extension step. Each PCR was performed in a volume of 50 μl as follows: 0.5 μg template DNA, 5 μl 10 × DreamTaq™ Buffer (20 mM Tris-HCl, pH 8.0; 1 mM DTT, 0.1 mM EDTA, 100 mM KCl, 0.5% Nonidet P40, 0.5% Tween 20, 50% glycerol), 5 μl 2.5 mM dNTP, 0.5 μl gene-specific primers (100 pmol/μl of each), 0.25 μl DreamTaq™ polymerase (5 U/μl, Fermentas, Glen Burnie, MD, USA), and nuclease-free water. DNA isolates from Ca Ski and HeLa cells infected with HPV16 and HPV18, respectively, were used as a positive control, and nuclease-free water was used as a negative control. Positive and negative controls were included with each run. The amplicons were separated using the QIAxcel DNA Screening Kit (Qiagen). The results were validated by direct sequencing of selected PCR products using the 96-capillary 3730xl DNA Analyzer (Applied Biosystems) to confirm the detected genotypes.

### Statistical analysis

GraphPad Prism 5.00 software (GraphPad Software, San Diego, CA, USA) was used for all analyses. Differences between groups were examined using the Fisher’s exact probability test according to the characteristics of data distribution. Logistic regression was used to calculate the ORs and their corresponding 95% CIs by comparing the case group to the control group. A *P*-value less than 0.05 was considered statistically significant. The number of samples (n) and the *P*-values of significant differences are given in tables.
